# ApoE Plasma Levels and Risk of Cardiovascular Mortality in Old Age

**DOI:** 10.1371/journal.pmed.0030176

**Published:** 2006-05-09

**Authors:** Simon P Mooijaart, Jimmy F. P Berbée, Diana van Heemst, Louis M Havekes, Anton J. M de Craen, P. Eline Slagboom, Patrick C. N Rensen, Rudi G. J Westendorp

**Affiliations:** **1**Department of Gerontology and Geriatrics, Leiden University Medical Center, Leiden, Netherlands; **2**Department of General Internal Medicine, Endocrinology and Metabolism, Leiden University Medical Center, Leiden, Netherlands; **3**Netherlands Organization for Applied Scientific Research—Quality of Life, Gaubius Laboratory, Leiden, Netherlands; **4**Department of Cardiology, Leiden University Medical Center, Leiden, Netherlands; **5**Section of Molecular Epidemiology, Leiden University Medical Center, Leiden, Netherlands; University of Utah Health Sciences CenterUnited States of America

## Abstract

**Background:**

The ɛ2, ɛ3, and ɛ4 alleles of the apolipoprotein E gene *(APOE)* encode three isoforms, apoE2, E3, and E4, respectively. The apoE isoforms circulate in different plasma concentrations, but plasma concentrations of the same isoform also differ between individuals. Whereas the isoforms have been associated with cardiovascular disease, the relation between plasma apoE levels and cardiovascular disease is unknown.

**Methods and Findings:**

We assessed *APOE* genotypes, plasma levels of apoE, cardiovascular risk factors, and mortality in a population-based sample of 546 individuals aged 85 y who participated in the Leiden 85-plus Study and were prospectively followed for specific causes of death for 5 y. Participants in the highest tertile of apoE levels suffered a twofold-increased risk of cardiovascular mortality (hazard ratio compared to lowest tertile, 2.08; 95% confidence interval [CI], 1.30 to 3.33). Among the 324 participants with the ɛ3ɛ3 genotype, the hazard from cardiovascular disease was threefold increased (highest versus lowest tertile 3.01; 95% CI 1.60 to 5.66), with similar estimates for men and women. Other causes of death were not increased significantly. Plasma levels of apoE in ɛ3ɛ3 participants were positively correlated with total cholesterol ( *p* < 0.001), low-density lipoprotein cholesterol ( *p* < 0.001) and triglycerides ( *p* < 0.001) and negatively with high-density lipoprotein cholesterol levels ( *p* = 0.010). Adjustment for plasma lipids did not change the hazard ratios, whereas interaction was absent. The risk associated with high levels of apoE, however, was strongest in participants from the lowest tertile of C-reactive protein (CRP) levels and absent in those from the highest tertile ( *p*_interaction_ < 0.001). Among participants from the lowest tertile of CRP levels, those with a high apoE levels had a significantly steeper increase in CRP than those with low apoE levels ( *p* = 0.020). Similar cardiovascular mortality risks as in ɛ3ɛ3 participants were found in ɛ2 and ɛ4 carriers.

**Conclusions:**

In old age, high plasma apoE levels precede an increase of circulating CRP and strongly associates with cardiovascular mortality, independent of *APOE* genotype and plasma lipids.

## Abstract 2

Among a cohort of people over 85 those with higher levels of apoE were more likely to die from cardiovascular disease. The observed association was independent of *APOE* genotype and plasma lipids.

## Introduction

The apolipoprotein E *(APOE)* gene influences lipid metabolism and disease risk. The encoded 299-residue plasma protein apoE is a surface component of primarily triglyceride-rich lipoproteins, such as very low-density lipoproteins (VLDLs), their remnants, chylomicron remnants, and high-density lipoproteins (HDLs). ApoE is the main ligand for clearance of VLDLs and chylomicron remnants, and as such affects circulating concentrations of lipoproteins and plasma levels of cholesterol and triglycerides [ 1].

The biological activity of apoE can be influenced by modification of its structure and/or quantity. A structural alteration arises from the two common ɛ2/ɛ3/ɛ4 polymorphisms [ 2], encoding apoE2, apoE3, and apoE4, respectively. ApoE2 exhibits lower affinity for the LDL receptor, resulting in slower clearance of apoE and higher plasma apoE levels [ 3, 4]. In response, the liver up-regulates the LDL receptor, resulting in lower cholesterol levels. Conversely, apoE4 is cleared more efficiently, resulting in lower apoE levels and higher cholesterol levels [ 3, 4]. The genetic variations thus affect lipid metabolism [ 5] and have been shown to alter risk of cardiovascular disease [ 6] and dementia [ 7]. Plasma apoE levels are only partially explained by the ɛ2/ɛ3/ɛ4 polymorphism [ 8], and plasma apoE levels vary between individuals with the same *APOE* genotype. Irrespective of *APOE* genotype, plasma apoE levels are also associated with cholesterol levels [ 9, 10]. Moreover, it was shown recently that apoE mediates the presentation of lipid antigens to the immune system and in this way influences the inflammatory process [ 11]. Both lipids and inflammation are involved in the pathogenesis of atherosclerosis, but the relation of plasma apoE levels and cardiovascular disease risk has not yet been reported.

Here, we studied the association of apoE plasma levels with cardiovascular mortality, independent of *APOE* genotype. To this end, within the Leiden 85-plus Study, a population-based prospective follow-up study, we determined the plasma levels of apoE, lipids, and C-reactive protein (CRP); *APOE* ɛ2/ɛ3/ɛ4 genotypes; and mortality from specific causes in old age.

## Methods

### Participants

Between 1 September 1997 and 1 September 1 1999, a total of 705 inhabitants of the community of Leiden, the Netherlands, reached the age of 85 y. Among these 85-y-old persons, we initiated a follow-up study to investigate determinants of successful aging [ 12]. There were no selection criteria on health or demographic characteristics. Fourteen inhabitants died before they could be enrolled. The response rate was 87%; a total of 599 persons (397 women and 202 men) participated [ 13]. There were no significant differences in various demographic characteristics between the 599 respondents and the source population. Of the 599 participants in the cohort, 38 refused to provide a blood sample, yielding a total number of 561 participants for the present study. The Medical Ethical Committee of the Leiden University Medical Center approved the study, and informed consent was obtained from all participants.

### Cardiovascular Disease History

History of myocardial infarction was obtained from the participants and from the treating physician. Information on use of medication was obtained from the participants' pharmacy records.

### *APOE* Genotypes

For genotyping, two TaqMan assays (Applied Biosystems, Foster City, California, United States) were used. For the single nucleotide polymorphism (SNP) in codon 112, primers were sense 5′- GCTGGGCGCGGACAT-3′ and antisense 5′- CACCTCGCCGCGGTACT-3′, and probes were 5′- CGGCCGCGCACGTCC-3′ labeled with FAM and 5′- AGGCGGCCGCACACGTC-3′ labeled with VIC. For the SNP in codon 158, an Assay-On-Demand (Applied Biosystems) was used (assay ID C____904973_10). The assays were run on a 7900HT (Applied Biosystems) according to manufacturer's specifications, with the following modifications: a Eurogentec qPCR core kit (Eurogentec, Seraing, Belgium) was used according to standard specifications; half the concentrations of primers and probes were used; the number of PCR cycles was 50. Fluorescence intensities were measured after the PCR, and genotypes were indicated by the Sequence Detection Software version 2.0 (Applied Biosystems).

### Plasma Measurements

At baseline, participants were visited twice at their places of residence within 1 mo after their 85th birthday. All blood samples were collected early in the morning, although not fasting. Within the 5 y after inclusion, participants were visited each year within a month of their birthday. Blood samples were drawn each year, and CRP and lipid levels were measured directly in fresh samples as described. ApoE levels were determined in 2005 in one batch of plasma samples that were collected at age 85 y at baseline of the study and stored frozen. Plasma apoE levels were determined using a human apoE-specific sandwich ELISA essentially as described [ 14]; an affinity-purified polyclonal sheep antihuman apoE antibody (ShαE/E, obtained by genetic immunization of sheep followed by boosting of animals with human apoE) was coated overnight onto MaxiSorp immune plates (Nunc Intermed, Roskilde, Denmark) (dilution 1:10 ^3^ in PBS [pH 7.4]; 100 μl/well) at 4 °C. Plates were washed three times with PBS containing 0.05% Tween-20 (v/v) (PBS-T), and unspecific binding sites were blocked for 1 h at 37 °C with blocking buffer (PBS containing 0.1% casein). Plates were washed three times with PBS-T, and 100 μL of the samples, reference sera (both dilution 1:8,000), and standards (plasma from C57Bl/6 mice spiked with 0–32 μg/l of human apoE) were added (all diluted in blocking buffer). The plates were incubated overnight at 37 °C. Plates were washed five times with PBS-T to remove unbound and/or nonspecifically bound proteins, and captured antigen was detected by adding 100 μL of horseradish peroxidase (HRP)-conjugated polyclonal sheep antihuman apoE antibody (dilution 1:10 ^3^ in blocking-buffer containing 0.05% Tween-20). After a 2 h incubation at 37 °C, plates were washed five times with PBS-T and 100 μl/well of freshly prepared tetramethylbenzidine (Organon Teknika, Boxtel, The Netherlands) was added, and the plates were put in the dark. After 20 min at room temperature the product formation was ended by addition of 100 μl/well of 2.5 M sulfuric acid. Following brief mixing, absorbance at 450 nm was measured. The inter-assay coefficient of variance was typically less than 10%, while the intra-assay coefficient of variance was typically less than 4%. Correlation coefficients of the calibration curves were typically better than 0.99. Reagent blanks had a typical absorbance of 0.06 (A450).

Plasma levels of total cholesterol, HDL cholesterol, triglycerides, and CRP were analyzed on fully automated computerized analyzers (Hitachi 747 and 911; Hitachi, Tokyo, Japan). The level of LDL cholesterol was estimated by the Friedewald equation (LDL cholesterol [mmol/l] = total cholesterol − HDL cholesterol − [triglycerides/2.2]), whereby participants with a triglyceride concentration higher than 443 mg/dl (5 mmol/l) were excluded ( *n* = 5).

### Other Cardiovascular Risk Factors

Participants were classified as having diabetes when they met at least one of the following criteria: (1) history of type 2 diabetes obtained from the general practitioner or treating physician; (2) use of sulfonylureas, biguanides, or insulin, based on information obtained from the participant's pharmacist; or (3) nonfasting glucose of 11.1 mmol/l or higher. Participants were classified as having hypertension when they met at least one of the following criteria: (1) history of hypertension obtained from the general practitioner or treating physician; or (2) mean systolic blood pressure of 165 mmHg or greater, or diastolic blood pressure of 95 mmHg or greater. Height and weight were measured at baseline, and body mass index (BMI) was calculated from these measurements. Self-reported history of smoking was categorized in three categories: never smoked, did smoke but not anymore, and current smokers.

### Causes of Death

For the analyses presented in this research, all participants were followed for mortality until 1 April 2004. During this period, of a total of 546 participants, 274 (50%) died. The date of death was obtained from the civic registries. Shortly after the civic registries reported the death of a participant, the general practitioner or nursing home physician was interviewed to determine the cause of death by means of a standardized questionnaire. Two senior specialists of internal medicine, unaware of the outcomes of the analyses, reviewed the causes of death and classified each death into primary causes of death according to the International Classification of Diseases and Related Health Problems, 10th Revision, as cardiovascular mortality (ICD codes I00–I99), infectious disease (ICD codes A00–B99 or J11–J18), cancer (ICD codes C00–D48), or other causes (all other ICD codes). Specific causes of death were not available for six participants.

### Statistical Analyses

Levels of total, LDL, and HDL cholesterol were normally distributed. Levels of plasma apoE, triglycerides, and CRP were not normally distributed, and are reported as geometric means. To allow transformation, undetectable levels of CRP were assigned the mean value 0.25. Differences in variables between groups were compared with a sex-adjusted linear regression. Sex-adjusted (geometrical) means and 95% confidence intervals (95% CIs), and differences between groups were calculated using a linear mixed model. Mortality risks were estimated using Cox proportional hazards model, and all were adjusted for gender. Prospective analysis of CRP levels was performed using a linear mixed model, adjusting for gender. All calculations were performed using SPSS 12.0.1 (SPSS, Chicago, Illinois, United States), and Kaplan-Meier curves were generated using S TATA 8 SE (Stata, College Station, Texas, United States) and replicated by two researchers independently (SPM, DvH).

## Results

### Baseline Study Population Characteristics

From the 561 eligible participants, measurement of plasma apoE failed in two and *APOE* genotyping failed in 13. The remaining 546 participants were included in the present analysis, of which the baseline characteristics are listed in Table 1. The *APOE* allele frequencies in our population were: ɛ2, 0.10; ɛ3, 0.77; and ɛ4, 0.13. The genotypes were in Hardy-Weinberg equilibrium and the allele frequencies were in line with comparable populations [ 10].

**Table 1:**
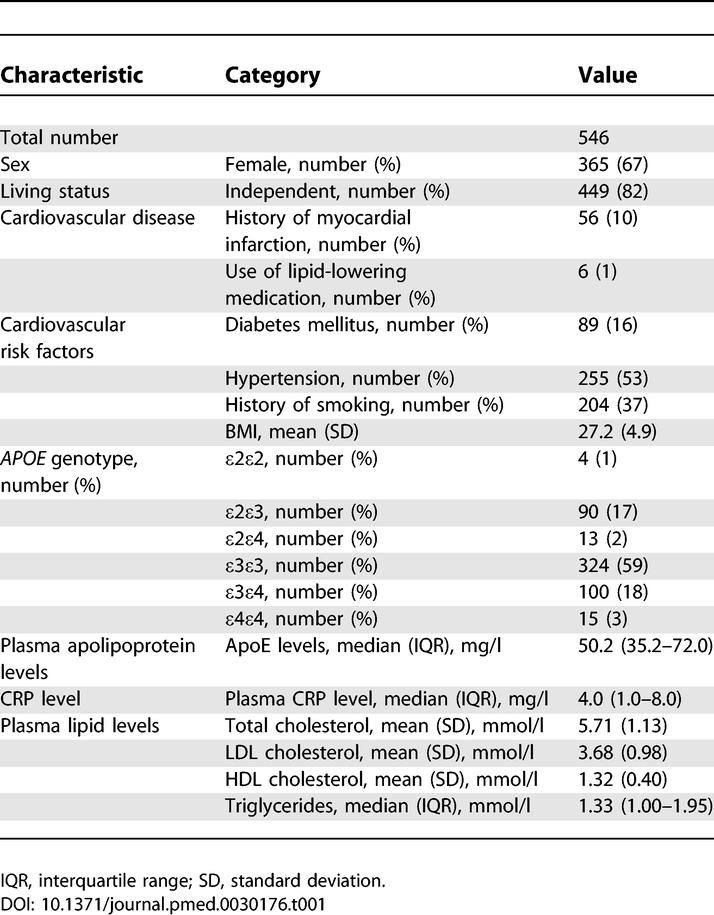
Baseline Characteristics of Study Participants

### Association of *APOE* Genotype with Plasma ApoE, CRP, and Lipid Levels at Baseline and Cardiovascular Mortality during Follow-Up

When assessing the relation of *APOE* genotype with plasma apoE levels, lipids, CRP levels, and cardiovascular mortality we found associations similar to those already described [ 3, 4, 9, 10]. In brief, participants who were carriers of the ɛ2 allele had significantly higher plasma levels of apoE and lower levels of LDL cholesterol and total cholesterol, whereas carriers of the ɛ4 allele had lower levels of plasma apoE and higher levels of LDL and total cholesterol ( Table 2). Levels of HDL cholesterol, triglycerides, and circulating CRP did not differ between the various genotypes. Compared to ɛ3ɛ3 participants, cardiovascular mortality was lower in ɛ2ɛ3 and ɛ2ɛ4 carriers, and higher in ɛ2ɛ2, ɛ3ɛ4, and ɛ4ɛ4 carriers, although none of the differences are statistically significant, possibly due to small numbers.

**Table 2:**
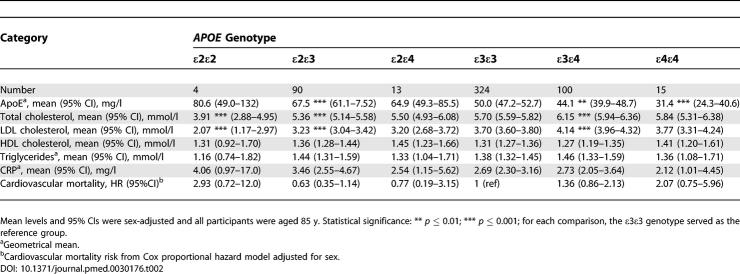
Plasma Levels of ApoE, Lipids, CRP, and Cardiovascular Mortality Risk Dependent on *APOE* Genotype

### Association of Plasma ApoE Levels and Mortality during Follow-Up

**Figure 1:**
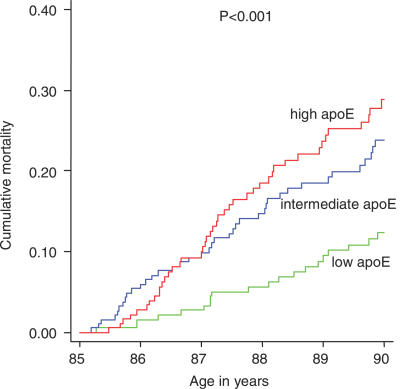
Kaplan-Meier Mortality Curve for Tertiles of ApoE Levels Plasma apoE is indicated at high (red), intermediate (blue) and low (green) levels, showing that high plasma apoE levels associate with increased cardiovascular mortality. The *p*-value indicates the statistical significance of the association of plasma apoE level with cardiovascular mortality in a sex-adjusted Cox proportional hazards model.

Mean follow-up time was 4.2 y. During follow-up, of a total of 546 participants, 274 (50%) died, of which 115 (42%) died of cardiovascular causes, 47 (17%) of infectious causes, 44 (16%) of cancer, and 62 (23%) of other causes. In the total study population, high apoE levels were associated with a 2.08-fold (95% CI, 1.30 to 3.33) increased risk, and intermediate levels with a 1.74-fold (95% CI, 1.09 to 2.79) increased risk, of cardiovascular mortality compared to those with low apoE levels ( Figure 1). This observation seems to contradict the associations described above, where the ɛ2ɛ3 genotype was associated with higher levels of apoE, lower LDL cholesterol, and a lower mortality risk, and ɛ3ɛ4 and ɛ4ɛ4 genotypes had lower levels of apoE, higher LDL cholesterol, and a higher mortality risk, albeit nonsignificantly.

To further investigate the effect of plasma apoE levels only and to eliminate the potentially distorting effect of structural changes in the apoE proteins, the relations between plasma levels of apoE and mortality were studied in ɛ3ɛ3 participants only. First, we assessed whether the effect of levels of apoE was specific for cardiovascular mortality as compared with other causes of death. Estimates of mortality risk dependent on plasma levels of apoE in ɛ3ɛ3 participants are listed in Table 3. Participants with high levels of plasma apoE had a twofold increased all-cause mortality risk (hazard ratio, 2.05; 95% CI, 1.39 to 3.02) as compared to those with low levels. This was predominantly due to a threefold increase in cardiovascular mortality risk (hazard ratio, 3.01; 95% CI, 1.60 to 5.66), whereas other causes of death were nonsignificantly increased. A similar increase in cardiovascular mortality was found in men (hazard ratio, 2.89; 95% CI 1.13 to 7.36) and women (hazard ratio, 2.96; 95% CI 1.27 to 6.90). Other causes of death were not increased significantly. When combined, mortality from all causes except cardiovascular disease was not significantly increased for participants with intermediate apoE levels (hazard ratio, 1.19; 95% CI 0.71 to 2.01) and high apoE levels (hazard ratio, 1.63; 95% CI 0.98 to 2.71), compared to participants with low levels. Second, we assessed whether the relation between levels of apoE and cardiovascular mortality risk was linear. When analyzing cardiovascular mortality risk over ten strata of plasma apoE level, we observed increasing cardiovascular mortality associated with increasing plasma apoE level in a gradual fashion (unpublished data).

**Table 3:**
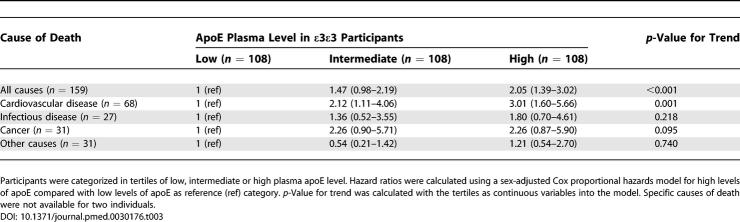
Mortality Risk according to Tertiles of Plasma Levels of ApoE in Participants with the ɛ3ɛ3 Genotype

### Association of Plasma ApoE Levels with Lipid Levels at Baseline and Relationship of Cardiovascular Mortality Risk Associated with Plasma ApoE and Lipid Levels

As expected, plasma levels of apoE strongly associated with levels of LDL and HDL cholesterol and triglycerides ( Table 4). Among ɛ3ɛ3 participants, those with high levels of apoE had significantly higher levels of total cholesterol ( *p* < 0.001), LDL cholesterol ( *p* < 0.001), and triglycerides ( *p* < 0.001) and lower HDL cholesterol ( *p* = 0.010). High levels of HDL cholesterol associated with decreased cardiovascular mortality compared to low levels (hazard ratio, 0.58; 95% CI, 0.36 to 0.92). No association was found with cardiovascular mortality for high levels of LDL cholesterol (hazard ratio, 0.89; 95% CI, 0.58 to 1.39) or triglycerides (hazard ratio, 1.00; 95% CI 0.65–1.56). To investigate whether the association of apoE levels with cardiovascular mortality was explained by an adverse lipid profile, we first analyzed the association of apoE with cardiovascular mortality in strata of low, middle, and high LDL and HDL cholesterol and triglycerides. We found that the risk associated with high level of apoE was similar in all tertiles of LDL and HDL cholesterol and triglycerides. Formal testing for interactions showed that there was no significant interaction between the risk of cardiovascular mortality associated with apoE and these lipids (all *p*_interaction_ > 0.6). We then adjusted the mortality analysis for levels of LDL and HDL cholesterol and triglycerides. Participants with high levels of apoE had mortality risks similar to those in the crude model (hazard ratio, 3.09; 95% CI, 1.55 to 6.15). Further adjustment for the cardiovascular risk factors hypertension, diabetes, BMI, and smoking did not change the estimate (hazard ratio, 3.20; 95% CI, 1.56 to 6.59). Finally, we repeated the cardiovascular mortality analysis within the ɛ3ɛ3 group, excluding individuals with a positive history of myocardial infarction ( *n* = 28) and those on lipid-lowering medication ( *n* = 4). In the fully adjusted model the risk associated with high levels of apoE was 4.14-fold higher than that of participants with low levels (95% CI, 1.75 to 9.84).

**Table 4:**
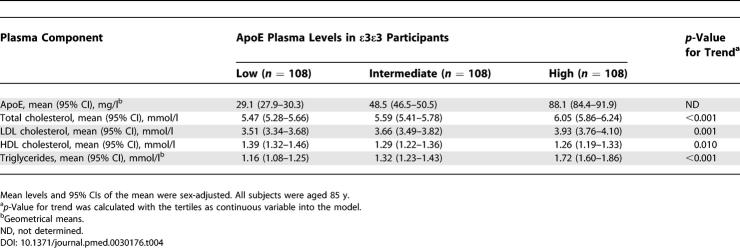
Plasma Levels of Total, LDL, and HDL Cholesterol and Triglycerides according to Tertiles Plasma Levels of ApoE in Participants with the ɛ3ɛ3 Genotype

### Association of Plasma ApoE Level and Increase in CRP Level during Follow-Up

**Figure 2:**
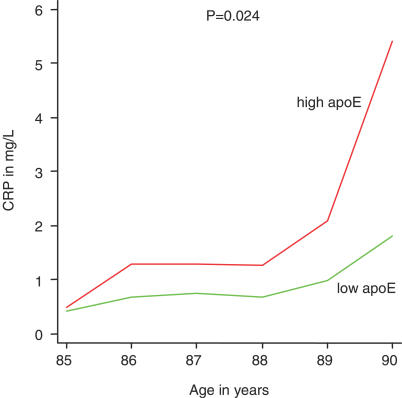
High versus Low Levels of Plasma ApoE High (red) and low (green) levels of the protein are depicted. Plasma apoE level was dichotomized on the median, showing that participants with high plasma apoE develop higher CRP levels during follow-up. Estimates of the geometric mean of circulating CRP levels for all years were calculated using a sex-adjusted linear mixed model. The *p*-value indicates the statistical significance of additional annual increases of CRP between high and low levels of apoE.

We also studied the risk of apoE in strata of CRP, another cardiovascular risk factor. In the total population, high levels of CRP are associated with increased cardiovascular mortality compared to low levels (hazard ratio, 2.42; 95% CI, 1.58 to 3.70). At baseline, ɛ3ɛ3 participants with high levels of apoE had significantly higher levels of CRP than those with intermediate and low levels ( *p*_trend_ < 0.001), and adjustment for sex and lipids did not alter the association ( *p*_trend_ < 0.001). The cardiovascular mortality risk associated with high levels of apoE was dependent on level of CRP ( Table 5), as it was highest in participants with low levels of CRP and absent in those with high levels of CRP ( *p*_interaction_ < 0.001). Adjustment for lipids ( *p*_interaction_ < 0.001) and other cardiovascular risk factors ( *p*_interaction_ < 0.001) did not alter the interaction. When participants with extreme levels of CRP (> 10 mg/l) were excluded from the analysis, the results remained similar. One possible interpretation of this interaction is that high levels of apoE precede an increase of CRP and in this way contribute to the pathogenesis of atherosclerosis. To test this hypothesis we investigated the association of baseline levels of apoE with levels of CRP during follow-up. We performed this analysis in the 91 participants in the lowest CRP stratum, because their risk of cardiovascular mortality associated with apoE was highest ( Table 5). Figure 2 shows the association of baseline levels of apoE with increases in CRP during follow-up. We found that high levels of apoE at baseline correlate with higher increases in levels of CRP during follow-up ( *p* = 0.024). Adjusting for classical cardiovascular risk factors did not change the observed association ( *p* = 0.020). A similar association was observed in ɛ4 carriers ( *p* = 0.041), but was absent in ɛ2 carriers ( *p* = 0.76).

**Table 5:**
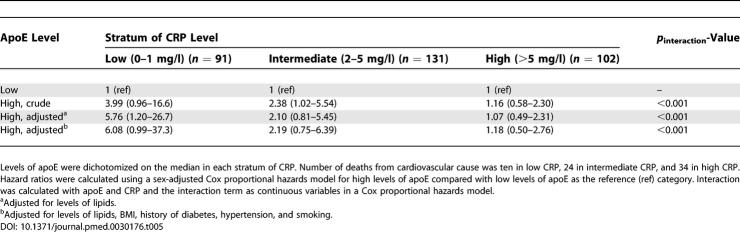
Cardiovascular Mortality Risk Associated with High Levels of ApoE in Strata of CRP in ɛ3ɛ3 Carriers

### Association of Plasma ApoE Levels and Cardiovascular Mortality in Different *APOE* Genotypes

To investigate whether the association of plasma apoE with cardiovascular mortality was also present in carriers of other genotypes, we repeated the mortality analysis in ɛ2 and ɛ4 carriers separately ( Table 6). We found a similar increase in cardiovascular mortality for participants in the highest apoE tertile as compared to the lowest tertile in ɛ2 carriers (hazard ratio, 2.43; 95% CI, 0.44 to 13.5) and ɛ4 carriers (hazard ratio, 4.74; 95% CI, 1.54 to 41.6). When the entire cohort was analyzed with *APOE* genotype accounted for, participants with high levels of apoE had a 3.45-fold increased risk of cardiovascular mortality (95% CI, 1.98 to 6.02).

**Table 6:**
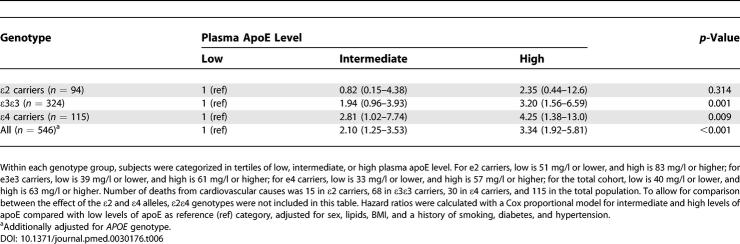
Cardiovascular Mortality Risk Dependent on Plasma ApoE Level in Subjects with Different *APOE* Genotypes

## Discussion

The main finding of our study is that in old age, individuals with high plasma levels of apoE are at an increased risk of cardiovascular mortality, independent of their *APOE* genotype, lipid levels, and other cardiovascular risk factors. In the prospective analysis, we also found that high plasma apoE levels precede a rise in CRP levels. Similar mortality risks were observed among carriers of the ɛ2 or ɛ4 allele.

### Plasma ApoE Levels Mark Proatherogenesis

ApoE is often referred to as an antiatherosclerotic protein (reviewed in [ 15]). This notion has its basis in observations in both mice and humans. Mice normally do not develop high LDL cholesterol levels or atherosclerosis, presumably because of higher VLDL turnover and the lack of cholesteryl ester transfer protein. Mice with genetically induced or diet-induced hyperlipidemia develop accelerated atherosclerosis that closely resembles all stages of human atherosclerosis, up to the stage of plaque formation. Although in mice plaque rupture does not occur and therefore no cardiovascular events occur, mouse models have proven valuable to study the biological mechanisms underlying the development of atherosclerosis in humans. In mice, complete knockout of the *apoe* gene causes hypercholesterolemia and early atherosclerosis [ 16]. Stem cell transplantation that enables these mice to produce small amounts of murine [ 17] or human [ 18] apoE in macrophages rescues them from atherosclerosis, whereas mice overexpressing rat apoE have a reduction in plasma lipoproteins and are resistant to diet-induced hypercholesterolemia [ 19]. In humans, a rare heritable deficiency of apoE also leads to the development of hyperlipoproteinemia and cardiovascular disease [ 20]. Within atherosclerotic lesions macrophages accumulate cholesterol and become foam cells, which play an important role in the initiation and progression of atherosclerosis [ 15]. ApoE is expressed by macrophages in atherosclerotic lesions in vessel walls [ 21] and up-regulates cholesterol efflux from macrophages [ 22]. In this way apoE is involved in decreasing the atherogenic effect of cholesterol. However, the main portion of the apoE plasma pool is produced by the liver and only a small fraction by macrophages [ 23], and plasma apoE levels are under tight genetic control [ 3, 24, 25]. This contrasts with apoE expression by macrophages, which is dependent on local factors, such as cholesterol loading and cytokines [ 15]. These findings suggest that plasma apoE levels may not correlate with macrophage apoE expression levels. Moreover, the antiatherosclerotic effect of apoE appears to be specific for macrophages, as transplantation of apoE-deficient macrophages into wild-type mice increases development of atherosclerosis, without affecting plasma levels of lipids and apoE [ 26]. Furthermore, we cannot exclude the possibility that expression of apoE in other tissues also leads to a reduction in atherosclerosis [ 27, 28]. These observations suggest that apoE plasma levels may have little relation to the antiatherosclerotic effect of apoE in macrophages.

### Proposed Mechanisms: Plasma ApoE Levels Associate with a Detrimental Lipoprotein Profile and/or with a Proinflammation Response

Here we show that high plasma apoE levels in fact do associate with an increased cardiovascular mortality risk. High plasma apoE levels may contribute to cardiovascular disease via one or both of two two hypothesized mechanisms. First, high levels of apoE may reflect a detrimental lipoprotein profile. We speculate that high apoE levels reflect the presence of higher levels of lipoprotein classes such as small, dense LDL, or VLDL and chylomicron remnants, which are proatherogenic [ 29, 30]. However, because we found in our analysis that the effect of apoE on cardiovascular mortality is independent of lipid levels, the effects of high apoE may not be mediated by LDL or HDL cholesterol or triglycerides per se. Levels of triglycerides are closely correlated with remnant concentrations [ 31], but did not associate with cardiovascular disease in our study [ 12, 32]. Furthermore, triglyceride levels do not contribute to the association of levels of apoE with cardiovascular disease mortality, as stratification and correction for triglyceride levels did not alter the effect of apoE levels on cardiovascular disease mortality. Changes in lipoprotein particle sizes have also been associated with longevity [ 33], suggesting that the association of apoE levels with cardiovascular mortality might be explained by changes in particle sizes. The standard fractionation method, which was used in the present study, is unable to distinguish between these subclasses. Alternative measurements of specific subclasses may help to elucidate the underlying detrimental lipid profile. Furthermore, measuring apoE concentrations in these subclasses could yield insight into which lipoprotein fraction contributes to the detrimental effect of high plasma apoE levels, or whether the detrimental effect of apoE is, for instance, specific for HDL-apoE or non-HDL apoE. However, since storage of plasma samples results in a shift of apoE between the different lipoproteins [ 34], and because a large portion of our study population has died, we are unable to perform such analysis on this cohort. We suggest that a separate study is initiated, including fractionation of fresh plasma, and measuring a series of apolipoproteins, including at least apoAI, apoB, and apoE within in these subclasses.

The second possible mechanism by which apoE contributes to cardiovascular disease may be related to the finding that apoE has proinflammatory properties. Plasma apoE avidly binds lipid antigens and appears to be critical for the presentation of lipid antigens by binding to antigen-presenting cells through the LDL receptor, which is followed by endocytosis [ 11]. The concomitant inflammatory response adequately eliminates the lipid antigen from the circulation. In this model, high plasma levels of apoE in combination with increased lipid-antigen presentation lead to chronic inflammation and thus may contribute to atherosclerosis. In line with these experimental data, we now show that high plasma apoE levels precede chronic inflammation (of which circulating levels of CRP are a marker [ 35]) and cardiovascular mortality risk. Taken together, we interpret these findings to suggest that, in old age, high plasma apoE levels associate better with a proinflammatory response and/or detrimental lipoprotein profile than with the antiatherosclerotic effect of apoE expression in macrophages.

### Plasma ApoE Levels Are a Cause, Not a Consequence, of Atherosclerosis

The association of apoE level and cardiovascular mortality provides new insight into mechanisms contributing to atherosclerosis. As the cardiovascular mortality risk associated with high levels of apoE was even higher in participants who did not have a history of myocardial infarction compared to those who did, high levels of apoE are unlikely to be the consequence of cardiovascular disease. In our study, the association of apoE levels with cardiovascular disease was strongest in participants with low levels of CRP, and in these persons high levels of apoE preceded an increase in CRP. However, in participants with high levels of CRP these associations were not observed. We interpret these results to indicate that high plasma apoE levels are an early and specific indicator of cardiovascular disease risk and drive a proinflammatory response. However, once there is a proinflammatory state—through whichever mechanism—high plasma apoE levels do not add to an increased risk. Taken together, our data may suggest that plasma apoE levels are a causal factor in cardiovascular mortality risk, and that at least part of the mechanism involves a proinflammatory response.

The observed association of high plasma apoE levels with increased inflammation seems to contradict earlier reports on specific anti-inflammatory properties of apoE. For instance, apoE was involved in the direct binding of lipopolysaccharide, the main toxic component of gram-negative bacteria [ 36], thereby decreasing the inflammatory response to lipopolysaccharide [ 37]. However, the pathways that neutralize endogenous and exogenous stimuli may be different. It is logical to assume such a pleiotropic effect, since genes regulating the inflammatory host response are under balanced evolutionary selection pressure.

### Literature Context

In our study population, as in other (younger) populations, apoE level associates with levels of total cholesterol, HDL cholesterol, LDL cholesterol, and triglycerides. In youth and middle age, high LDL cholesterol and low HDL cholesterol are strong risk factors of cardiovascular disease. In contrast, in old age, only levels of HDL cholesterol, but not of total cholesterol, LDL cholesterol, and triglycerides, affect cardiovascular disease risk [ 12, 32, 38, 39]. It is tempting to speculate that a high apoE plasma level is an independent risk factor of cardiovascular disease in middle age as it is in old age.

We found, as in other (younger) study populations, that *APOE* genotypes associate with plasma levels of apoE. Plasma apoE levels are highly dependent on heritable factors [ 3, 24, 25], but the *APOE* ɛ2/ɛ3/ɛ4 genotype is only a minor determinant of these levels [ 8, 40], and accordingly the plasma levels of apoE varied markedly in persons with ɛ3ɛ3 genotypes. ApoE2 has lower receptor binding affinity than apoE3 and apoE4 [ 41]. ApoE4 binds to lipid particles with higher affinity than does apoE3 and shows preference for VLDL, whereas apoE2 and apoE3 mainly bind to HDL [ 42]. The results of individual studies on the association of the ɛ2 allele and cardiovascular disease have been conflicting. Some studies report significant, both harmful and protective associations, whereas others have reported no associations [ 43]. On the other hand, the ɛ4 allele has been associated with an increased risk of cardiovascular disease in a meta-analysis of studies up to 1996 [ 43]. Here we show that the effect of the plasma levels of apoE masks the effect of the common *APOE* ɛ2/ɛ3/ɛ4 genotypes and may explain why the reported associations of the *APOE* genotype have been contradictory; the effect of genotype should be adjusted for plasma apoE levels.

### Strengths and Weaknesses of the Present Study

Blood samples were not drawn fasting, which could add to the random error of the measurement of triglycerides, apoE, and CRP. However, all samples were drawn in the early morning, and the observed associations of *APOE* genotype with plasma apoE level, and of apoE plasma level with triglyceride level, were similar to those found in other studies using fasting samples, which suggests that the magnitude of random error is relatively small. The low number of participants on lipid-lowering medication may result from the restrictive medication policy by Dutch general practitioners at the time of the enrollment of the Leiden 85-plus Study (1997–1999). This restrictive policy has been one of the motivations to initiate a clinical trial. The PROSPER study, in part carried out in the same region as the current study, showed that lipid-lowering medication (in this case pravastatin) protects individuals from coronary artery disease at least up to the age of 82 [ 38]. However, the results of this study were published several years after the start of the Leiden 85-plus Study (2003). CRP levels were not measured with high-sensitivity methods. The use of high-sensitivity CRP measurement is especially informative in discriminating between samples with none or very little CRP. However, in this age group the number of participants with levels of CRP below the detection limit of the assay was relatively small (102 out of 563 participants), and the variation in CRP levels was considerable. Furthermore, most of the analyses performed with these CRP levels involved stratification for CRP, and it is unlikely that high-sensitivity measurements would have systematically changed the attrition of participants to these strata. Therefore we believe that the measurement that we used resulted in adequate discriminative power.

A limitation of our study is that this finding cannot directly be extrapolated to the incidence of nonfatal cardiovascular disease, and not beyond this age group. The current study was performed in a population-based sample of 85-y-olds. The participants of this study were the survivors of their birth cohort, and earlier studies in people of over 70 y of age have shown that biology in old age may be different from that in other age groups, which may prevent extrapolation of the results to the population at large. For instance, LDL cholesterol is no longer a cardiovascular risk factor [ 38]. Reaching the age of 85 y is becoming less unusual than is generally expected. The proportion of the original birth cohort that has reached this age is 36% for women and 15% for men, and this proportion will further increase in the future. Furthermore, to our knowledge we are the first to report an association of plasma apoE levels with cardiovascular mortality in any age group, and it is tempting to speculate that this finding holds in other age groups. A strength of our study is the specificity and sensitivity of the analyses due to the high incidence of fatal events during follow-up. Moreover, the data come from a population-based study without inclusion criteria based on health and demographic characteristics. Another strong point is that we could restrict our analyses to *APOE* ɛ3ɛ3 carriers, thereby eliminating the potentially distorting effect of structural changes in the apoE protein.

### General Conclusion

The present data show that a high apoE plasma level is a new predictor of cardiovascular mortality in elderly people. We conclude that levels of apoE may indicate a detrimental lipoprotein profile and/or a proinflammatory response, which both contribute to cardiovascular mortality.

## Supporting Information

### Accession Numbers

The GenBank ( http://www.ncbi.nlm.nih.gov) accession numbers for apolipoprotein E are as follows: *APOE* codon 112 (rs429358), *APOE* codon 158 (rs7412), *APOE* mRNA (NM_000041), and apoE protein (NP_000032).

**Editors' Summary**

**Background.**

Atherosclerosis is a common disorder of the blood vessels. Blood lipids (fatty substances, for example cholesterol and other substances collect on the inner walls of the vessels and form layers, known as plaques. Eventually, these plaques can interfere with blood flow though the blood vessels. They can also break apart, causing debris to travel through the bloodstream to other parts of the body and block vessels there. This rupture is a common cause of heart attack and stroke. Scientists believe that inflammation (that is, activation of the body's immune system) helps cause atherosclerosis. ApoE is a protein present in the blood that influences how cholesterol and other fats are made and removed from the body. ApoE can also have an effect on inflammation. Different people have different levels of apoE in their blood, depending on which version of the *APOE* gene (which codes for the apoE protein) they have as well as other factors.

**Why Was This Study Done?**

Knowing that apoE can influence both blood lipid levels and inflammation, and that the specific version of the *APOE* gene can affect a person's risk for cardiovascular disease, this study was designed to test whether blood levels of apoE can then affect the risk of death from cardiovascular disease (such as heart attack and stroke).

**What Did the Researchers Do and Find?**

They studied 546 patients who were 85 years and older. All patients gave blood samples within a month after their 85 ^th^ birthday and then every following year within a month of their birthday. Within the five years of the study, 274 participants died, 115 of them from cardiovascular disease. The researchers analyzed the blood samples in the following ways. They measured apoE protein levels in the first blood sample and also determined the variants of the *APOE* gene for each participant. For the first and all following yearly blood samples, they also measured cholesterol, other lipids, and a substance called CRP. CRP is a marker of inflammation and known to be raised in patients with cardiovascular disease. The researchers found that in these patients, those who had high levels of apoE at age 85 had a higher risk of later death from cardiovascular disease. This higher risk was independent of which version of the *APOE* gene the patients had. It was also independent of their lipid levels and other known risk factors such as smoking and diabetes. The strongest relationship between apoE levels and cardiovascular mortality was seen in patients who had low CRP levels at age 85. In most of these patients, CRP levels rose in the following years, suggesting that high apoE levels come before an increase in inflammation.

**What Do These Findings Mean?**

In old age, at least, high apoE concentrations in the blood indicate an increased risk of death from cardiovascular disease. The results suggest that the common assumption that apoE has antiatherosclerotic effects and therefore protects against cardiovascular disease is too simple. ApoE levels in the blood might have different effects than apoE in specialized immune cells called macrophages that are involved in atherosclerosis. Only six of the 546 participants were taking lipid-lowering drugs such as statins. This is a much smaller percentage than would be expected under current recommendations in the US and Europe. It is not clear whether the same results would have been found if more of the patients had been taking other lipid-lowering drugs, so more research is needed to study whether the observed relationship also holds for persons on lipid-lowering medication.

**Additional Information.**

Please access these Web sites via the online version of this summary at http://dx.doi.org/10.1371/journal.pmed.0030176.

• Medline Plus pages for heart disease

• Information on heart and vascular diseases from the US National Heart, Lung, and Blood Institute

• Patient information sheets from the American Heart Association

• Patient information on lipoproteins from the US National Lipid Association

## Abbreviations

ApoE: apolipoprotein E
BMI: body mass index
CI: confidence interval
CRP: C-reactive protein
HDL: high-density lipoprotein
LDL: low-density lipoprotein
VLDL: very low-density lipoprotein
